# Control of Wnt Receptor Turnover by R-spondin-ZNRF3/RNF43 Signaling Module and Its Dysregulation in Cancer

**DOI:** 10.3390/cancers8060054

**Published:** 2016-06-08

**Authors:** Huai-Xiang Hao, Xiaomo Jiang, Feng Cong

**Affiliations:** Novartis Institutes for Biomedical Research, 181 Massachusetts Avenue, Cambridge, MA 02139, USA; huaixiang.hao@novartis.com (H.-X.H.); xiaomo.jiang@novartis.com (X.J.)

**Keywords:** Wnt signaling pathway, targeted therapy, RNF43, ZNRF3, R-spondin, Frizzled

## Abstract

Aberrant activation of the Wnt/β-catenin pathway is frequently found in various cancers, often through mutations of downstream components. Inhibiting β-catenin signaling in tumors with downstream pathway mutations remains challenging, due to a lack of favorable targets. On the other hand, targeting upstream components of the Wnt pathway is rather straightforward. However, it is difficult to identify tumors addicted to autocrine or paracrine Wnt signaling. Discovery of the R-spondin-ZNRF3/RNF43 signaling module and its genetic alterations in cancers represents a breakthrough in this area. Membrane E3 ligase ZNRF3 and RNF43 are critical negative feedback regulators of the Wnt pathway, which function through promoting ubiquitination and degradation of Wnt receptors. R-spondin proteins (RSPO1-4) serve as natural antagonists of ZNRF3/RNF43. To maintain strong and sustained Wnt/β-catenin signaling, cancers need to overcome ZNRF3/RNF43-mediated feedback inhibition. Indeed, mutations of RNF43/ZNRF3 and recurrent translocations of RSPO2/RSPO3 have recently been identified in various cancers. Significantly, genetic alterations in RNF43/ZNRF3/RSPO2/RSPO3 have shown promise as predictive biomarkers in pre-clinical models for the efficacy of upstream Wnt inhibitors. In this review, we will discuss the biology of the R-spondin-ZNRF3/RNF43 signaling module, cancer-associated alterations of this signaling module, and their value as biomarkers to identify Wnt-addicted tumors.

## 1. Introduction

The evolutionarily conserved Wnt signaling pathway plays critical roles in embryonic development and adult tissue homeostasis in all multicellular animals [1]. Wnt proteins are secreted lipoglycoprotein ligands that control cell proliferation, migration, cell fate specification, and polarity formation. The canonical Wnt signaling cascade drives specific gene expression programs through regulating the stability of transcription cofactor β-catenin. Wnt proteins can also activate the β-catenin–independent Plana Cell Polarity (PCP) pathway to coordinate cell and tissue movements. The Frizzled (FZD) family of seven transmembrane-domain proteins serves as the core receptors of Wnt proteins, and they are required for both Wnt/β-catenin and Wnt/PCP signaling. Wnt proteins utilize different coreceptors to activate different downstream signaling pathways; Wnt proteins bind to coreceptor LRP5/6 to turn on the Wnt/β-catenin pathway, and they bind to coreceptor ROR1/2, RYK or PTK7 to initiate the Wnt/PCP pathway. The strength of Wnt signaling is tightly regulated and several feedback regulatory mechanisms control proper signaling output. Aberrant Wnt signaling is associated with various diseases including cancer.

## 2. ZNRF3 and RNF43 Regulate Wnt Receptor Turnover

**Figure 1:**
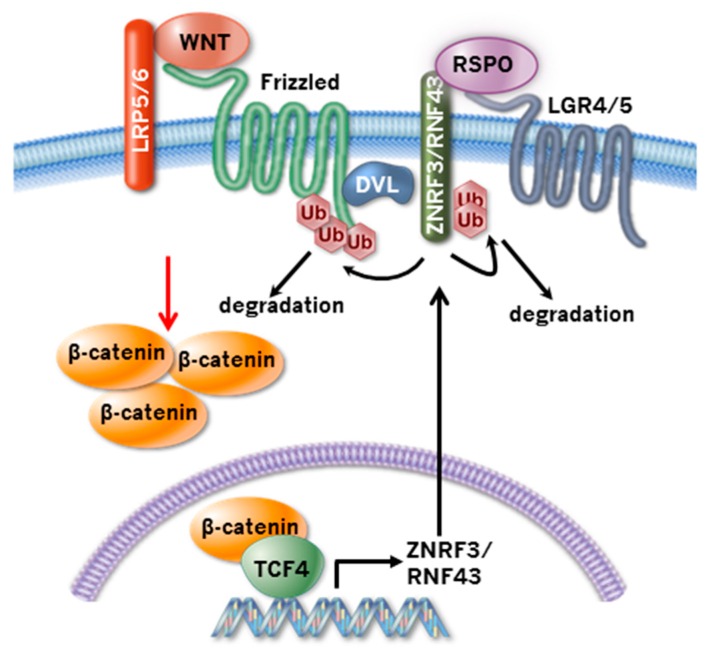
Feedback control of Wnt receptor turnover by R-spondin-ZNRF3/RNF43 signaling module. Wnt proteins interact with FZD and LRP5/6 to initiate Wnt/β-catenin signaling. Stabilized β-catenin enters the nucleus, binds to TCF family transcription factors, and induces the expression of ZNRF3/RNF43. ZNRF3 and RNF43 translocate to the plasma membrane, recognize FZD through DVL, and induce ubiquitination and degradation of FZD. This shuts off Wnt/β-catenin signaling. The function of ZNRF3/RNF43 is counteracted by R-spondin; R-spondin binds to LGR4/5 and ZNRF3/RNF43 and induces ubiquitination and degradation of ZNRF3/RNF43. To achieve high and sustained Wnt/β-catenin signaling, cancer cells need to overcome this strong negative feedback control, which can be achieved through mutations of RNF43/ZNRF3 or translocations/overexpression of R-spondin.

Ubiquitination-mediated turnover of Wnt receptors has emerged as a critical regulatory mechanism of Wnt pathway activity as it determines the responsiveness of cells to Wnt ligands. Cell surface FZD levels are stabilized by UBPY/USP8 [2] and USP6 [3], suggesting that ubiquitination serves as an important regulatory mechanism underlying FZD lysosomal degradation. Since feedback control is an important feature of the Wnt signaling pathway, people have studied β-catenin target genes to search for novel Wnt pathway regulators. Using this approach, our lab and Clever’s lab independently identified cell surface transmembrane E3 ubiquitin ligase Zinc and Ring Finger 3 (ZNRF3) and its functional homolog Ring finger protein 43 (RNF43) as negative feedback regulators of Wnt signaling [4,5]. ZNRF3 and RNF43 are related to the Goliath family of transmembrane RING domain E3 ligases, and have a unique structural organization. They have a signal peptide, an extracellular domain, a transmembrane domain, and an intracellular RING domain. ZNRF3 and RNF43 inhibit Wnt/β-catenin signaling through promoting ubiquitination and subsequent internalization and degradation of Wnt receptor FZD and LRP6 [4,5] (Figure 1). The *C. elegans* protein PLR-1, ortholog of ZNRF3 and RNF43, also regulates Wnt receptor turnover, suggesting that this function is evolutionarily conserved [6]. The function of ZNRF3 and RNF43 in Wnt signaling is supported by mouse genetic studies. Knockout of Znrf3 blocks lens development through inducing Wnt/β-catenin signaling in the lens placode [4]. Double knockout of Znrf3 and Rnf43 in mouse intestine induce strong expansion of the intestinal stem cell zone and rapid formation of intestine adenoma [5]. Consistent with a critical role of FZD in Wnt/PCP signaling, ZNRF3 and RNF43 also control the Wnt/PCP signaling pathway [4]. Znrf3 knockout embryos often show neural tube closure defects, which are associated with defective PCP signaling. Overexpression of either wild-type or catalytic dead mutant of ZNRF3 disrupts PCP signaling and causes convergent extension defects in zebrafish embryos.

The molecular mechanism by which ZNRF3 and RNF43 recognize FZD has been elucidated [7]. Dishevelled (DVL) serves as a positive regulator of Wnt signaling through directly binding to FZD and promoting clustering of Wnt receptors [8]. Recent studies revealed an unexpected role of DVL in promoting Wnt receptor degradation. DVL is found to be associated with ZNRF3/RNF43 and DVL knockout cells show significantly elevated FZD cell surface expression and decreased FZD ubiquitination. These results suggest that DVL serves as an adaptor protein targeting ZNRF3/RNF43 to FZD to promote FZD ubiquitination and degradation.

## 3. R-spondin-ZNRF3/RNF43 Signaling Module

R-spondin proteins (RSPO1-4) are secreted proteins that potently sensitize cells to Wnt/β-catenin signaling and Wnt/PCP signaling [9,10]. All four R-spondin proteins have similar domain structures with two N-terminal Furin domains and a C-terminal TSR domain. Two Furin domains are necessary and sufficient to activate both Wnt/β-catenin and Wnt/PCP signaling [11,12,13,14]. LGR4 and LGR5, members of the Rhodopsin G-protein coupled receptor (GPCR) family, are high affinity receptors of R-spondin; R-spondin requires LGR4/5 to activate Wnt signaling, but it does not activate canonical GPCR signaling downstream of LGR4/5 [11,15,16,17]. Discovery of ZNRF3/RNF43 and the finding that R-spondin increases the cell surface levels of FZD have led to elucidation of the molecular mechanism by which R-spondin and LGR4/5 potentiate the Wnt pathway [4] (Figure 1). R-spondin simultaneously binds to the extracellular domains of ZNRF3/RNF43 and LGR4/5, and induces auto-ubiquitination and membrane clearance of ZNRF3/RNF43, resulting in increased cell surface level of FZD. Regulation of FZD turnover explains how R-spondin can control both Wnt/β-catenin and Wnt/PCP signaling. This molecular model is supported by co-crystal structure and mutational analysis of R-spondin-LGR4/5-ZNRF3/RNF43 complexes [18,19,20,21,22,23,24]. R-spondin binds to LGR4/5 through the Furin 2 domain, and binds to ZNRF3/RNF43 through the Furin 1 domain. R-spondin needs to interact with both LGR4/5 and ZNRF3/RNF43 to be functional; mutations disrupting either interaction completely abolish the Wnt stimulatory activity of R-spondin. In this complex, LGR4/5 functions as the engagement receptor while ZNRF3/RNF43 functions as the efficacy receptor for R-spondin. Wnt stimulatory activities of different R-spondin proteins are correlated with their binding affinities to ZNRF3 or RNF43. In cell-based Wnt reporter assays, RSPO2 and RSPO3 are more potent than RSPO1 and RSPO4, and RSPO4 is the least active RSPO [13,24]. Consistently, RSPO2 and RSPO3 bind to ZNRF3 with nanomolar affinity, RSPO1 and RSPO4 bind to ZNRF3 with micromolar affinity, and RSPO4 has the weakest binding to ZNRF3 [18,24,25].

R-spondin proteins are important for diverse biological processes such as sex determination, vasculature formation, and development of limb, lung, hair, nail, and muscle [12,26,27,28,29,30], most likely through potentiating Wnt signaling. The physiological role of the R-spondin-ZNRF3/RNF43 signaling module is best studied in the self-renewal of digestive tract epithelium, where proliferation is driven by Wnt/β-catenin signaling. Since ZNRF3 and RNF43 are β-catenin target genes, they are coexpressed in intestinal stem cells which have the highest level of β-catenin signaling. In these cells, ZNRF3 and RNF43 impose a strong negative feedback control of Wnt/β-catenin signaling and prevent over-amplification of intestinal stem cells. Knockout of Znrf3 and Rnf43 in mouse intestinal epithelium causes unrestricted expansion of the intestinal stem cell zone [5]. Consistent with the notion that R-spondin functions as a natural antagonist of ZNRF3/RNF43, systemic overexpression of R-spondin induces a strong expansion of intestinal crypts [31]. In the physiological condition, the negative function of ZNRF3 and RNF43 on Wnt signaling in intestinal stem cells appears to be counter-balanced by stromal cell-derived R-spondin. Knockout of R-spondin receptor Lgr4/5 in the intestinal epithelium abolishes the stem cell compartment and causes the rapid demise of intestinal crypts [16,32]. Depletion of both RSPO2 and RSPO3 using neutralizing antibodies abolishes regeneration of the intestinal epithelium upon irradiation [33]. Intestinal stem cells can be cultured in matrigel to initiate self-organizing, continuously propagating organoids that contain all known types of differentiated cells [34], and R-spondin constitutes an essential component of this culture system. Since this culture system is devoid of stromal cells, it is consistent with the idea that stromal cell-derived R-spondin is critical for the self-renewal of intestinal stem cells. Thus, the R-spondin-ZNRF3/RNF43 signaling module represents an elegant system to fine-tune Wnt/β-catenin signaling and ensure proper self-renewal and differentiation of intestinal stem cells. A similar mechanism probably also exists in other tissue stem cells.

## 4. RNF43/ZNRF3 Mutations and RSPO2/3 Translocations in Cancer

Aberrant activation of Wnt/β-catenin signaling has long been implicated in cancer [1]. Several downstream components of the Wnt pathway are frequently mutated in colorectal cancer and various other tumors. These include truncation mutations of APC, stabilization mutations of β-catenin and loss/gain-of-function mutations of AXIN1 [35]. Expression changes of Wnt ligands and secreted Wnt pathway inhibitors have also been observed in cancer [36]. Recent studies suggest that the R-spondin-ZNRF3/RNF43 signaling module is frequently disrupted in cancer.

### 4.1. RNF43/ZNRF3 Mutations

The initial report of RNF43 mutations was in benign pancreatic tumors called intraductal papillary mucinous neoplasm (IPMN) and mucinous cystic neoplasm (MCN) [37]. With the broader adoption of next generation sequencing, mutations of RNF43 were subsequently identified in a variety of cancers including colorectal adenocarcinomas [38], endometrial carcinomas [38], mucinous ovarian carcinoma [39], pancreatic cancer [40,41], and gastric cancer [42] at frequencies ranging from 4.0% to 18.9% (Table 1). Mutation of ZNRF3 in cancer is less frequent as compared with RNF43 mutations. Adrenocortical carcinoma (ACC) is the only cancer type with a frequent mutation of ZNRF3 at 21.0% [43]. In fact, ZNFR3 is the most frequently altered gene in ACC. It is unclear why RNF43 is more susceptible to mutations than ZNRF3. This difference of mutation rates might be attributed to the stability of different DNA regions or the expression levels/activities of these two proteins.

With the completion of most large-scale sequencing studies of common cancer types (e.g., The Cancer Genome Atlas, TCGA), the tumor types listed in Table 1 are likely to represent all the common cancers with frequent mutations (>5.0%) in RNF43 and ZNRF3. Many of these cancers, in particular colon cancer, gastric cancer and endometrial tumor, are well known for aberrant Wnt signaling activation, evidenced by the prevalence of APC mutations or β-catenin mutations [35]. As expected, RNF43 and ZNRF3 mutations tend to be mutually exclusive with APC and β-catenin mutations in those tumor types [38,43], reflecting their shared consequences in activating the Wnt/β-catenin signaling pathway. However, not all the cancer types previously associated with downstream Wnt pathway mutations harbor RNF43/ZNRF3 mutations. For example, there was no report of RNF43 or ZNRF3 mutations in hepatocellular carcinoma, where AXIN1 and β-catenin mutations occur in nearly half of the cases [44]. This is in contrast to the 3.5%~9.3% RNF43 mutations in a closely related type of liver cancer, cholangiocarcinoma, depending on the association or lack thereof with a parasite called liver fluke [45,46]. Interestingly, mutations of APC, AXIN1, or β-catenin are also rare in cholangiocarcinoma [45,46]. This extreme example of two subtypes of liver cancer suggests that different tumor types may utilize different mutational mechanisms to activate the β-catenin pathway or require different levels of β-catenin signaling for optimal cell proliferation. The frequency of RNF43 mutations in melanoma, breast cancer, or head and neck malignancies is lower than 2%.

Identified RNF43 mutations are mostly truncating events (non-sense mutations and frame-shift mutations) and reported missense mutations spread across the gene. This is consistent with the tumor suppressor role of RNF43. There are two recurrent hotspot mutations: G659fs and R117fs. They account for 41.7%~48.0% and 8.3%~12.0% of RNF43 mutations identified in colon cancer and endometrial cancer [38]. These two mutations are close to the microsatellite instability (MSI) loci. This may explain why cancers with a deficiency in DNA mismatch repair, such as colon cancer, endometrial cancer and gastric cancer, have frequent RNF43 mutations. Indeed, the MSI subtype of gastric cancer has a nearly 10-fold higher mutation frequency in RNF43 than that of the microsatellite stable (MSS) subtype (54.6% *versus* 4.8%, Table 1). RNF43 mutation frequency is 79.7% in the MSI subtype of colorectal cancer and 50.7% in the MSI subtype of endometrial cancer. One can argue that RNF43 G659fs or R117fs mutations might not be driver mutations as they are mainly found in tumors with high mutation burden. However, according to an algorithm called InVEx, these two mutations emerge much more frequently than one would expect under a model of no selection, suggesting that they confer a fitness advantage for colorectal and endometrial cancer cells [38].

### 4.2. RSPO2/3 Translocations

In just one year after the initial discovery of RNF43 mutations in IPMN and MCN, recurrent RSPO2 and RSPO3 translocations were identified in 3.0% and 8.0% of colon cancer, respectively [47]. Translocations were discovered by RNA sequencing of 68 colon tumors and both translocations presented in the MSS subtype of colon cancer. The 5’ fusion partners are EIF3E and PTPRK, respectively. There are at least three configurations of the fusion transcripts: EIF3E(e1)–RSPO2(e2), PTPRK(e1)–RSPO3(e2) and PTPRK(e7)–RSPO3(e2) (e stands for exon). Fusion transcripts encode functional RSPO2/3 proteins under the control of stronger EIF3E promoter or PTPRK promoter. Indeed, the expression of RSPO2 and RSPO3 in colon tumor samples containing such fusions is significantly elevated compared with tumor samples without the fusions. In addition, RSPO2/3 translocations are mutually exclusive with APC or β-catenin mutations, indicating that they probably have a similar role in the activation of Wnt/β-catenin signaling. Similar EIF3E-RSPO2 and PTPRK-RSPO3 fusions are also reported in 4.0% of 75 Japanese colorectal cancer samples [48]. Although the frequency is lower than that reported in the previous study [47], the Japanese colorectal cancer samples with RSPO2/3 fusions are similarly positive for the expression of mismatch repair proteins and have the wild-type APC allele. Taken together, RSPO2/3 translocations and RNF43 mutations are enriched in distinct subtypes of colorectal cancer in terms of mismatch repair. Recently, high frequencies of PTPRK-RSPO3 fusions (31%) and RNF43 mutations (24%) have been reported in colorectal traditional serrated adenomas (TSAs) [49].

Interestingly, RSPO2/3 translocations have also been identified in other tumor types. Recurrent RSPO2 and RSPO3 fusions are found in prostate cancers and Schwannoma [50,51]. In addition, a group from Janssen reported RSPO2 and RSPO3 translocations in 3.0% of 324 non-small cell lung cancer (NSCLC) samples (197 squamous subtype and 127 adenocarcinoma subtype) [52]. Interestingly, all the fusion transcripts were detected only in the squamous subtype of NSCLC. Common genetic alterations in NSCLC such as mutations in EGFR, ALK and KRAS are much more frequent in the non-squamous subtype, which has benefited the most from the existence of new therapies directed to those targets. If verified, RSPO2/3 translocations are likely driver mutations and represent one of the first druggable alterations in the squamous subtype of NSCLC with high unmet medical need. The fact that only RSPO2/3 translocations are identified in tumors could be related with the higher Wnt-amplifying activity of these two isoforms.

## 5. Function of R-spondin-ZNRF3/RNF43 in Tumor Biology

ZNRF3/RNF43 represents a powerful negative feedback mechanism identified in the Wnt pathway. Activation of Wnt/β-catenin signaling induces the expression of ZNRF3 and RNF43, which in turn promote the degradation of Wnt receptors on the cell surface and shut down Wnt signaling. The strength of this negative feedback regulation is demonstrated by the finding that the knockdown of β?-catenin in a pancreatic cancer line with wild-type ZNRF3 and RNF43 strongly increases the cell surface level of FZD, most likely through down-regulation of endogenous RNF43 and ZNRF3 [53]. Therefore, to achieve high and sustained Wnt/β-catenin signaling, it would be critical for cancer to bypass this potent negative feedback regulation. This can be achieved through the mutation of RNF43/ZNRF3 or overexpression of R-spondin.

Mouse genetic studies have supported a critical role of the R-spondin-ZNRF3/RNF43 signaling module in cancer. Rspo2 and Rspo3 were identified as sites of integration for mouse mammary tumor virus (MMTV)-induced mammary tumors [54,55,56]. Rspo2 was identified as a colorectal cancer candidate gene in a transposon-based genetic screen in mice [57]. Double knockout of Znrf3/Rnf43 induces rapid formation of intestinal adenoma [5] and these tumors are strictly Wnt-dependent [58]. In addition, proliferation of RNF43-mutated pancreatic cancers is suppressed by the re-expression of wild-type RNF43 [53]. Collectively, these data suggest that RSPO2/3 fusions and RNF43/ZNRF3 mutations are oncogenic events.

As discussed previously, R-spondin enhances both Wnt/β-catenin signaling and Wnt/PCP signaling through increasing the cell surface level of FZD. Wnt/PCP signaling has long been associated with cell movement, invasion and tumor metastasis [36]. Although R-spondin-induced Wnt/β-catenin signaling clearly plays a major role in driving tumorigenesis, the importance of R-spondin-induced Wnt/PCP signaling in cancer should not be overlooked. For example, although Rspo2 and Wnt1 are both found as common integration sites in the MMTV-induced mouse mammary tumor model, Rspo2- and Wnt1-transformed mammary epithelial cells behave differently *in vivo* [59]. As compared with Wnt1 tumors, Rspo2 tumors exhibit an epithelia-mesenchymal transformation (EMT) phenotype and have greater metastatic activity. Importantly, the invasive properties of Rspo2-expressing cells cannot be blocked by DKK1, a specific inhibitor of Wnt/β-catenin signaling. These results suggest that Rspo2-induced cell invasion is most likely mediated by Wnt/PCP signaling. A potential role of R-spondin-induced Wnt/PCP signaling in cancer invasion/metastasis is also reported in lung cancer [60]. RSPO3 overexpression is found in KEAP1-deficient lung adenocarcinoma, and it is associated with poor survival. Knockdown of RSPO3 or its receptor LGR4 reverses EMT and decreases cell migration *in vitro* and inhibits metastasis *in vivo*.

Our understanding of the R-spondin-ZNRF3/RNF43 signaling module in cancer is still evolving. An alternative mechanism of the RNF43-mediated Wnt pathway inhibition has recently been proposed [61]. In this model, RNF43 is located on the nuclear membrane and it tethers TCF4 to the nuclear membrane, thus silencing TCF4 transcriptional activity. This would position RNF43 downstream of β-catenin, rather than upstream of β-catenin. The significance of this finding is not clear since RNF43 mutations and APC mutations in colorectal cancer are mutually exclusive and the localization of RNF43 on the inner layer of the nuclear membrane has not been demonstrated. RNF43 has a signal peptide and a transmembrane domain, so RNF43 might be located on the outer layer of the nuclear membrane, which is continuous to the ER membrane. It should be noted that R-spondin might have a tumor-suppressor function. RSPO1 appears to function as a tumor suppressor in skin carcinoma [30], suggesting that the function of Wnt/RSPO signaling in cancer could be context-dependent. It has also been proposed that RSPO2-LGR5 signaling has tumor-suppressive activity in colorectal cancer with APC mutations [62]. It is shown that RSPO2 is silenced in colorectal cancer due to promoter hypermethylation, and that exogenous RSPO2 suppresses β-catenin signaling through stabilization of ZNRF3 on the plasma membrane. It is currently unclear how membrane ZNRF3 can block β-catenin signaling in colon cancer cells with β-catenin mutation or APC truncation. Clearly, additional studies are needed to corroborate these findings and to fully understand the function of the R-spondin-ZNRF3/RNF43 signaling module in tumor initiation, maintenance, and metastasis in various tumor types.

## 6. RNF43/ZNRF3 Mutations and RSPO2/RSPO3 Translocations as Biomarkers to Identify Wnt-Dependent Tumors

Multiple upstream Wnt inhibitors have been generated, including pan-FZD antibodies [63], LRP6 antibodies [64,65], decoy FZD receptor [66], and porcupine inhibitors [67,68,69]. Readers should refer to a recent review [70] for a comprehensive overview of experimental therapeutics targeting the Wnt pathway. Before identification of the R-spondin-ZNRF3/RNF43 signaling module, the patient selection strategies for these Wnt signaling inhibitors had been mostly limited to expression biomarkers that are usually less robust. Identification of cancer cell lines dependent on autocrine Wnt signaling *in vitro* is not trivial. In the past, people often used overexpression of secreted Wnt inhibitor DKK1 or sFRPs to demonstrate Wnt dependency. However, using this approach to demonstrate Wnt dependency could be complicated since overexpression of DKK1 or sFRPs can cause ER stress and yield false-positive data. Porcupine is a membrane-bound *O*-acyltransferase that is specifically required for the palmitoylation of Wnt ligands, a necessary step in the secretion of Wnt ligands. Identification of WNT974, formerly known as LGK974, as a highly potent and selective small-molecule porcupine inhibitor has significantly improved our ability to identify Wnt-dependent tumors [67]. We tested WNT974-induced inhibition of β-catenin target gene expression and WNT974-induced growth inhibition in 39 pancreatic cell lines [53]. Although clear down-regulation of β-catenin target genes by WNT974, an indication of active autocrine Wnt/β-catenin signaling, is observed in most cell lines, only three cell lines show strong proliferation inhibition following WNT974 treatment. Importantly, all three cell lines have loss of function mutations of RNF43 with loss of the wild-type allele. In xenograft models, WNT974 inhibits proliferation and induces differentiation of RNF43 mutant pancreatic tumors. It should be noted that RNF43 mutant tumors depend on the Wnt pathway even in the presence of KRAS mutations that are nearly universal in pancreatic cancer. This study represents the first example of using RNF43 mutations as a biomarker to identify Wnt-dependent tumors. The predictive value of RNF43 mutations for Wnt inhibition has been further demonstrated using a structurally distinct porcupine inhibitor named ETC-1922159 [69].

It should also be noted that RNF43 mutant tumors can be Wnt-independent. PATU8988S and PATU8988T are two pancreatic cancer cell lines derived from the same patient. Although both cell lines have the same RNF43 mutation, only PATU8988S is sensitive to porcupine inhibitor [53]. Similarly, in two colon cancer organoids derived from the same patient (MSI subtype), only the organoid with the RNF43 G355fs mutation but not the one with the RNF43 G659fs mutation responds to the porcupine inhibitor *in vitro* [71].

Identification of RSPO2/RSPO3 translocations in cancer provides not only an attractive target for Wnt pathway inhibition using antibody approaches but also an intuitive predictive biomarker for RSPO2/RSPO3 antibodies. Since R-spondin sensitizes cells to Wnt ligands through inhibiting ZNRF3/RNF43, tumor cells harboring RSPO2/RSPO3 translocations should be sensitive to either direction inhibition of RSPO2/RSPO3 or inhibition of Wnt ligand secretion by porcupine inhibitors. Indeed, colorectal cancers with RSPO3 fusion are sensitive to RSPO3 antibody [33] and porcupine inhibitor [69]. In addition, overexpression of R-spondin is found in a variety of solid tumors through translocation-independent mechanisms, and such tumors are sensitive to R-spondin antibodies in mouse xenograft models [72]. Beyond R-spondin-neutralizing antibodies, there could be other strategies to block R-spondin signaling, such as extracellular domains of LGR4/5 as decoy receptors or LGR4/5 antibodies that block R-spondin-LGR4/5 interaction. Blocking R-spondin signaling in cancer using such strategies is not reported yet.

In summary, RNF43 mutations and R-spondin translocation might serve as predicative biomarkers to identify Wnt-dependent tumors. Both RNF43 mutations and RSPO fusions are currently being used as patient enrollment criteria in the clinical trial of porcupine inhibitor WNT974 (ClinicalTrials.gov, NCT02278133). The value of using such biomarkers for patient selection will be determined.

## 7. Conclusions and Perspectives

The regulation of Wnt receptor turnover by the RSPO-ZNRF3/RNF43 signaling module has emerged as a key regulatory mechanism of Wnt signaling. Dysregulation of this signaling module is frequent in cancer. To maintain high and sustained Wnt/β-catenin signaling, cancer cells must inactivate this negative feedback mechanism, which can be achieved through the mutation of ZNRF3/RNF43 or overexpression of R-spondin. Importantly, such cancers are likely addicted to autocrine or paracrine Wnt signaling and sensitive to upstream Wnt inhibitors such as porcupine inhibitors and FZD antibodies. RNF43/ZNRF3 mutations and R-spondin translocation/overexpression can be used as biomarkers to identify Wnt-dependent tumors for administering upstream Wnt inhibitors in clinical trials.

One major issue of using Wnt inhibitors is on-target toxicity due to the importance of Wnt signaling in several tissues such as the bone and the gut. Predictive biomarkers such as RNF43/ZNRF3 mutations and RSPO2/RSPO3 translocations serve to identify tumors exquisitely sensitive to Wnt inhibition. Administrating Wnt inhibitors to patients harboring RNF43/ZNRF3 mutations or RSPO2/RSPO3 translocations in their tumors may result in a wider therapeutic window and enable the successful clinical development of Wnt inhibitors. In addition, due to the functional redundancy of four R-spondin proteins, R-spondin antibodies might have a bigger therapeutic window than general Wnt pathway inhibitors such as porcupine inhibitors and pan-FZD antibodies in tumors with R-spondin translocation/overexpression. Another important consideration is combination strategy. Although Wnt inhibitors show single agent activities in preclinical models, stronger anti-tumor response is seen when Wnt inhibitors are combined with chemotherapeutic agents such as taxol [63,72]. Depending on the co-occurring oncogenic mutations, combinations with other targeted therapies such as EGFR inhibitors should also be considered. Future studies are needed to refine patient selection criteria and drug combination strategy to achieve deeper and more prolonged anti-tumor response and to reduce on-target toxicity of Wnt inhibitors.

## Figures and Tables

**Table 1:** *RNF43* mutations in different cancer types.

Cancers	Mutation Frequency	Sample Size	Ref.	Comments
Intraductal papillary mucinous neoplasm (IPMN) and mucinous cystic neoplasm (MCN)	75% 37.5%	8 IPMN 8 MCN	[37]	These tumors can progress to pancreatic ductal adenocarcinoma.
Cholangiocarcinoma (CCA) *O. viverrini* associated Non-*O. viverrini* associated	9.3% 3.5%	54 86	[45,46]	
Mucinous ovarian carcinomas	21%	29	[39]	2/22 (9%) in mucinous ovarian borderline tumors.
Gastric cancer Microsatellite-stable (MSS) microsatellite instability (MSI)	4.8% 54.6%	100	[42]	Recurrent G659fs mutations in MSI subtype
Colorectal adenocarcinomas NHS and HPFS dataset TCGA dataset	18.9% 17.6%	185 224	[38]	Recurrent G659fs mutations and R117fs mutations.
Endometrial carcinomas	18.1%	248	[38]	Recurrent G659fs mutations
Pancreatic carcinomas with acinar differentiation	4%	23	[40]	
Pancreatic cancer	6%	109	[41]	

The only cancer type with frequent ZNRF3 mutations is adrenocortical carcinomas (ACC) at 21% from a 45 sample study [43]. NHS: Nurses’ Health Study; HPFS: Health Professionals Health Professionals Follow-Up Study; TCGA: The Cancer Genome Atlas.
